# Health promotion interventions for increasing stroke awareness in ethnic minorities: a systematic review of the literature

**DOI:** 10.1186/1471-2458-14-409

**Published:** 2014-04-28

**Authors:** Paolo Gardois, Andrew Booth, Elizabeth Goyder, Tony Ryan

**Affiliations:** 1 School of Health and Related Research, University of Sheffield, Regent Court, 30 Regent Street, Sheffield S1 4DA, UK; 2 School of Nursing and Midwifery, The University of Sheffield, Barber House, 387 Glossop Road, Sheffield S10 2HQ, UK

## Abstract

**Background:**

Stroke places a significant burden to all affected individuals, but it is perhaps more significant amongst members of black, minority and ethnic communities, who may experience poorer awareness of stroke symptoms than the general population. Recently, several initiatives tried to improve public awareness that symptoms of stroke need to be treated as a medical emergency. However, ethnic communities present cultural barriers, requiring tailored health promotion interventions, whose effectiveness remains uncertain. Our systematic review aimed to identify relevant published evidence, synthesize the main study components and identify evidence of the effectiveness of the interventions.

**Methods:**

MEDLINE, EMBASE, CINAHL, and PsycInfo were searched for journal articles on health promotion interventions for increasing stroke awareness in ethnic minorities, published in English between 1995 and 2012. Search results were collaboratively assessed by the authors; included studies were analysed to identify their main characteristics, and a thematic analysis of their content was conducted. No meta-analysis was performed, due to the heterogeneity of results.

**Results:**

Eighteen studies were included, reporting 15 interventions conducted in the US, for African-Americans or Hispanics; populations sizes differed between interventions. Interventions were mostly carried out in community settings with different educational techniques, focussing on experiential methods. Health professionals usually organized the programs, delivered by nurses, other health professionals or volunteers.

The few theory-based interventions focussed on individual-level behavioural change. Practical cultural adaptation strategies were not linked to specific theoretical frameworks. Interventions widely differed as for target populations, settings, delivery methods, contents and professional roles involved. All study designs were quantitative, and the emerging evidence of effectiveness was inconclusive.

Such interventions operate in very complex scenarios, and several variables may influence their effectiveness. Therefore, qualitative or mixed-methods study designs may shed light on barriers and facilitators, experiential education strategies and community involvement.

Network- and community-level theories may help improving design and evaluation of interventions.

**Conclusions:**

Eleven case reports and four RCTs provide evidence about stroke awareness interventions organized in the US. The studies provide only partial and inconclusive evidence about the effectiveness of the interventions. Hence, further research is needed on different countries and ethnic minorities.

## Background

The need to increase stroke awareness in black, minority and ethnic communities (BMEs) is a difficult, but vital challenge, required to save lives and reduce inequalities.

Stroke places a significant burden on BME community members. The second highest cause of death in the world [1], stroke in 2004 had a worldwide prevalence of 30.7 million cases, with 9 million new cases every year [2]. A significant proportion of patients die from stroke [3], and survivors often experience disability or impairment for US data, see [4,5]. Consequently, health system costs resulting from stroke-related deaths and disabilities are high, between 2 and 4% of total expenditure [3,6,7]. This significant financial burden is likely to increase in the future since the majority of strokes affects the elderly [7]. Studies from the U.S. [8,9] and the UK [10,11] suggest that ethnic minorities experience higher stroke risk and incidence and worse outcomes in comparison to the general population, due to hereditary, environmental, social and health-system factors. Moreover, ethnic minorities experience increased risk of pre-hospital delays, and show lower stroke awareness than the general population.

Current evidence based management of stroke involves rtPA thrombolysis within three hours from onset [12]. It is thus imperative that symptoms are recognized early to allow prompt admission to the nearest stroke unit. Nonetheless, very few eligible patients receive the recommended treatment [13,14], mainly because of delayed hospital admission due to pre-hospital delays, i.e. delays occurring from the onset of symptoms to admission [15,16]. Two studies suggests that belonging to a BME community increases the risk of significant pre-hospital delays [16,17].

In addition to acting on preventable risk factors [18], the strategy of increasing stroke awareness has often been employed to reduce pre-hospital delays, both in the general population and in BME communities. A recent review showed that awareness of symptoms of stroke is low in the general population, and tends to be poorer in ethnic communities [19]. Consequently, stroke awareness improvement is part of national stroke strategies in different countries [20-23]. Furthermore, a recent review [16] shows that initiatives to improve awareness that symptoms of stroke need to be treated as a medical emergency have recently been undertaken in several countries. Such initiatives used different channels and strategies, such as mass media advertisements, community involvement and health education techniques. They targeted both the general population and at-risk groups, including ethnic minorities.

However, the evidence of effectiveness of the initiatives for stroke awareness improvement is inconclusive, especially for interventions targeting BME populations. Awareness of stroke symptoms does not automatically translate into the ability to recognize such stroke symptoms in a patient [24]. In particular, no linear relationship emerges between the level of stroke awareness in a community, the behavioural intent to call emergency medical services (EMS) when witnessing a stroke, and the reduction of pre-hospital delays [25-27]. Deciding to call the EMS when witnessing a stroke resembles a collective, network-mediated, community-based decision-making process [28-30]. Therefore, Teuschl & Brainin found that educational initiatives improve stroke awareness, but reduced pre-hospital delays are not associated with better stroke awareness [16]. Ethnic communities include a high concentration of at-risk individuals, and present cultural barriers to the uptake of stroke awareness messages, requiring community-based and culturally tailored health promotion interventions [31,32]. For example, in the UK the effect of mass media campaigns such as F.A.S.T.^a^[33] on BME community members seems to be limited [34,35]. Thisprompted the UK National Audit Office to state that "The Department [of Health] (…) should consider particularly how to engage with groups at higher risk of stroke, such as people of Afro-Caribbean and South Asian ethnicity" [36]. Finally, recent contributions have tried to define the cultural appropriateness of interventions and the main principles for conducting research in ethnicity and health [37-39].

It is therefore critical to understand which health promotion interventions have been carried out to increase awareness of stroke symptoms and adequate response in BME communities, and whether there is evidence to demonstrate their effectiveness. Consequently, we decided to review the literature to establish the main characteristics of health promotion interventions directed towards increasing awareness of stroke symptoms and appropriate response (i.e., calling EMS) in BME communities. Additionally, we assessed the evidence of their effectiveness.

## Methods

To answer our research questions, we undertook a systematic review of the literature. We aimed to retrieve all studies describing health promotion interventions designed for increasing awareness of stroke symptoms and appropriate response (i.e., calling EMS) in BME communities. We also decided to map the main study components and to identify any evidence of effectiveness of the described interventions. This section describes the search strategy, inclusion and exclusion criteria for identified articles, the process of article selection and the process of data analysis and synthesis.

Our review had the following objectives:

identifying relevant published evidence;

selecting studies according to rigorous inclusion and exclusion criteria;

synthesizing the main components of included studies;

identifying evidence of the effectiveness of the described interventions, if any.

The PRISMA checklist [40] was used as a guide to report the results of the review.

### Search, screening and selection strategy

Firstly, we conducted a systematic search on four health sciences databases: Pubmed MEDLINE, EMBASE, CINAHL, and PsycInfo. The searches were performed on 17/01/2012, with the search string described in Table 1.

**Table 1:** Search strings used in the Pubmed database

**Steps**	**Search string**
1	("Stroke"[Mesh] AND (knowledge OR ("warning sign" OR "warning signs") OR recognition OR awareness) Limits: Humans, English, Publication Date from 1996)
2	(stroke AND (knowledge OR ("warning sign" OR "warning signs") OR recognition OR awareness) AND ("2011/07/15"[Date - Entrez] : "3000" [Date - Entrez]))
3	1 OR 2

The string matched our inclusion and exclusion criteria (see below), and it was agreed upon by all authors, following pilot searches that combined different permutations of terms. The second and third steps in the search strategy were directed at identifying In-Process articles included in the database in the six months prior to the search, which would be lost if using only the first step in the strategy. The first author performed the same search, with the necessary adaptations, in the other three databases.

### Inclusion and exclusion criteria

Only studies describing one or more health promotion interventions aimed at increasing awareness of stroke symptoms, warning signs and appropriate response (i.e., calling EMS) in BME communities were eligible for inclusion. We defined BME communities as any ethnic minority community in any country. Consequently, any intervention whose target audience was composed of at least 50% BME community members was eligible for inclusion. We included interventions if they targeted either the general population of BME communities, or specific subcategories, e.g. students, parents, the elderly or stroke survivors. However, we excluded articles describing health interventions only aimed at health professionals or lay health workers, including health champions (i.e. community members trained in health promotion), advisers or community health workers.

We also included studies if stroke awareness was not the sole aim of the reported interventions. For example, some articles related to interventions concerning stroke risk factors in addition to stroke awareness, while others concerned knowledge of cardiovascular diseases or diabetes alongside stroke awareness itself. We excluded studies identifying barriers, facilitators, or specific attitudes and needs of potential targets of stroke awareness interventions, and studies identifying theoretical issues not related to data emerging from health promotion interventions.

Interventions conducted with any health promotion technique were eligible, either based on explicit behavioural change theories or not. We did not exclude any article because of the study design. However, we only included refereed articles since they usually represent the most updated and highest-quality literature on health promotion, in comparison to other scientific contributions (e.g.: books, conference proceedings, dissertations, etc.). We only included articles in English for practical reasons relating to non-availability of translation services. In addition, we considered eligible for inclusion only articles published from 1996, since the first trial showing effectiveness and safety of rtPA for stroke treatment if administered within three hours of onset of symptoms was published in December 1995 [41]. It is therefore assumed that this trial would only have had an impact on health promotion interventions after 1995.

### Data analysis and synthesis

A first scan reading of the included articles allowed us to produce a first group of categories used to summarize the content of the articles. We then developed a sheet [42], using Microsoft Excel 2010. The sheet was refined while reading the full text of the articles and through discussion between authors. In its final form, it included the columns now showed in Tables 2, 3, 4 and 5.

**Table 2:** Characteristics of populations targeted by the interventions

**ID**	**Study**	**Target ethnic groups**	**Intervention level**	**Population**	**Population size**
1	Boden-Albala 2010 [44]	Hispanics; African Americans	Groups	Survivors (stroke and TIA)	Large: 736
2	Chan 2008 [45]	African Americans	Individuals	General population	Medium: 198 1
3	Covington 2010 [46]	African Americans	Groups	General population	Small: 16
4	Dromerick 2011 [47]	African Americans	Individuals	Survivors (stroke and TIA)	Medium: 250
5	Duraski 2003 [48]; Duraski 2006 [49]	Hispanics	Groups	General population	Medium: 177
6	Duraski 2007 [50]	Hispanics	Groups	Children and young adults (aged 9–26)	Small: 32
7	Frank 2008 [51]	African Americans	Groups	Parishioners of African-American churches	Medium: 120
8	Kalenderian 2009 [53]	African Americans; Hispanics	Groups	Individuals taking part in church activities	Large: > 1500
9	Kleindorfer 2008 [54]	African Americans	Individuals	Women	Medium: 383
10	Miller 2003 [55]	African Americans	Individuals	Patients at risk for stroke	Small: 60
11	Morgenstern 2007 [56]; Gonzales 2007 [52]; Mullen Conley 2010 [29]	Mexican Americans	Groups	Middle school students and their parents	Large: 706
12	Villablanca 2009 [57]	African Americans; Hispanics	Groups	Women aged > 40 years	Large: 1052
13	Williams 2008 [58]	Hispanics; African Americans	Groups	Students aged 9-11	Large: 582
14	Williams 2012 [59]	African Americans; Hispanics	Individuals	Parents of primary school children	Medium: 101
15	Williamson 2009 [60]	African Americans	Groups	Members of an Afro-American church	Medium: 325

**Table 3:** Outcomes and study design of selected studies

**ID**	**Study**	**Study design**	**Evaluation method**	**Reported effectiveness**^**a**^
1	Boden Albala 2010 [44]	Randomized controlled trial (RCT)	Article reports only on protocol and baseline	Article reports only on protocol and baseline
2	Chan 2008 [45]	RCT	Pre-post test	Yes
3	Covington 2010 [46]	Case study	None	Not applicable
4	Dromerick 2011 [47]	RCT	Article reports only on protocol and baseline	Article reports only on protocol and baseline
5	Duraski 2006 [49]; Duraski 2003 [48]	Case study	Pre-post test	Yes
6	Duraski 2007 [50]	Case study	None	Not applicable
7	Frank 2008 [51]	Case study	Pre-post test	No
8	Kalenderian 2009 [53]	Case study	None	Not applicable
9	Kleindorfer 2008 [54]	Case study	Pre-post test	Yes
10	Miller 2003 [55]	Case study (repeated measures design with 3 groups)	Pre-post test	No effectiveness for treatment seeking behaviour (call EMS); unknown effectiveness for knowledge of stroke symptoms
11	Morgenstern 2007 [56]; Gonzales 2007 [52]; Mullen Conley 2010 [29]	RCT	Pre-post test	Yes for children; unknown for parents
12	Villablanca 2009 [57]	Case study	None (only for outcomes other than stroke symptoms)	Not applicable
13	Williams 2008 [58]	Case study	Pre-post test	Yes
14	Williams 2012 [59]	Case study	Pre-post test	Yes
15	Williamson 2009 [60]	Case study	None	Not applicable

^a^Effectiveness of an intervention refers to its ability to improve the knowledge of stroke symptoms and the intention to call 999 in target populations.

**Table 4:** Intervention type, focus, duration and setting

**ID**	**Study**	**Intervention type**	**Focus**	**Duration of intervention**	**Setting**
1	Boden-Albala 2010 [44]	• Two sessions about stroke education	Awareness	2 brief sessions within 3 weeks of stroke/TIA onset	Hospital or home
2	Chan 2008 [45]	• Stroke education program (video)	Awareness	12 minutes	Emergency department
3	Covington 2010 [46]	• PowerPoint presentation	Equal focus	Single, brief session	• Churches
		• Educational materials to take home			• Group homes
		• Blood pressure screening and referral			• Community centers, and community organizations"
4	Dromerick 2011 [47]	• Stroke navigators visiting patients	Equal focus	Advice sessions over one year	Home
5	Duraski 2006 [49]; Duraski 2003 [48]	• Short slide presentation	Equal focus	1 to 2 hours	Community centres and community organizations
		• Stroke risk assessment screening			
		• Advice/discussion.			
6	Duraski 2007 [50]	• Focus group session	Awareness	30 to 60 minutes	Unknown
		• Slide presentation			
		• Interactive questions/answers			
7	Frank 2008 [51]	• Cardiovascular diseases and stroke education sessions	Prevention/risk factors	About 2 hours for each intervention	African-American churches
		• Screening			
		• Integration with Bible study, individual counselling, healthy food			
8	Kalenderian 2009 [53]	• Educational sessions, distribution of educational package to "ambassadors"	Prevention/risk factors	Various, depending on specific interventions	Faith-based institutions, churches
		• Educational activities by ambassadors in churches, e.g. by brochures, videos, posters.			
9	Kleindorfer 2008 [54]	• Trained beauticians educated their customers	Awareness	A session at the beauty salon	Beauty salons
		• Distribution of stroke-related study packets			
10	Miller 2003 [55]	• Education about knowledge of stroke symptoms and modifiable stroke risk factors.	Equal focus	1-hour initial educational intervention:15’ follow-up	Medical practice (some follow-ups at home).
11	Morgenstern 2007 [56]; Gonzales 2007 [52]; Mullen Conley 2010 [29]	• Lessons to children about stroke signs and symptoms and to improve skills, self-efficacy and behaviour.	Awareness	• Four 50-minute classes each year for three years	School and home
		• Parents were taught about stroke by their children as homework assignment		• homework with parents at home.	
12	Villablanca 2009 [57]	• Clinical lectures	Prevention/risk factors	12-14 counselling sessions, (only a minority on stroke awareness)	Various faith-based, academic and non-academic sites
		• Health demonstrations, video presentations, personal testimonies, medical screenings			
13	Williams 2008 [58]	• "Culturally and age-appropriate music and dance to enhance an interactive didactic curriculum including the FAST mnemonic"	Awareness	1-hour sessions over 3 consecutive days	School
14	Williams 2012 [59]	• Stroke communication intervention	Equal focus	Short (not quantified)	Home
		• Shared completion of stroke-related homework between children and parents			
15	Williamson 2009 [60]	• "Educational session	Prevention/risk factors	Interventions over two years	A rural African American church
		• Health screenings and weight watchers program			
		• Integration with faith-based activities			

**Table 5:** Health professionals, theories and cultural adaptation of interventions

**ID**	**Study**	**Administered by**	**Theories**	**Cultural adaptation**
1	Boden-Albala 2010 [44]	• Two health educators	• Social cognitive theory	• Bilingual materials with translation by community health worker
		• 1 physician or nurse	• Motivational interviewing	• Visuals integrating community places
				• Film footage of community stroke survivors recalling stroke experiences in their own language
				• Integration and instructions for current community resources
				• Conversations about barriers such as mistrust of the health care system
				• A community committee evaluated cultural appropriateness of the intervention
				• Involvement of local stroke support group
2	Chan 2008 [45]	• African American actors instructed by Stroke Association	• None	• Video produced by the American Heart Association, with African-American actors
3	Covington 2010 [46]	• Trained college students acting as health champions	• Social cognitive theory	• Generic mention that the presentations were "culturally sensitive".
			• Stages of change	
4	Dromerick 2011 [47]	• Lay community health workers	• Theory of reasoned action	• Usage of American Heart Association’s tailored educational materials
			• theory of planned behaviour	• Provision of tailored health education
			• motivational interviewing	
5	Duraski 2006 [49]; Duraski 2003 [48]	• Research nurse	• None	• Presentation developed for the Hispanic culture
				• Emphasis on risk factors affecting the Hispanic community
				• Information was not literally translated to Spanish".
				• Verbal/written educational materials in Spanish about stroke warning signs/symptoms
				• Focus groups with communities to ensure appropriateness of presentation
6	Duraski 2007 [50]	• Research nurse	• None	• Option to have focus groups in Spanish or English
				• Culturally sensitive information, not simply translated from English to Spanish
7	Frank 2008 [51]	Nurse researchers	• None	• No
		• Nursing students		
8	Kalenderian 2009 [53]	Trained ambassadors	• None	• No
9	Kleindorfer 2008 [54]	•	• None	• No
10	Miller 2003 [55]	• Neuroscience nurses	• Stages of change	• No
			• Motivational interviewing	
11	Morgenstern 2007 [56]; Gonzales 2007 [52]; Mullen Conley 2010 [59]	• Educator	• Social cognitive theory	• Culturally sensitive strategy developed through a focus group with parents, students and teachers."
		• Stroke neurologist		
		• Data manager		• Aspects of Mexican-American culture included inclusion of Mexican American health professionals in design
		• Science/health teachers		
		• KIDS project health professionals		• Focus groups with local students, parents and teachers;.bilingual materials
12	Villablanca 2009 [57]	• Site leaders	• Stages of change	• Culturally appropriate health education curriculum and materials
		• Cardiologists		
		• Endocrinologists		
		• Nurses		
		• Dietitians		
		• Physical exercise and other health professionals"		
13	Williams 2008 [58]	• Two stroke education professionals	• None	• Rap and hip-hop
		• 2 community health professionals		
14	Williams 2012 [59]	• Children administered the intervention	• Theory of reasoned action	• Rap and hip-hop (songs and dance)
			• Social cognitive theory (self-efficacy)	
15	Williamson 2009 [60]	• Nurses	• None	• No
		• Nursing students		

Given the heterogeneity of the included study designs, a quantitative meta-analysis of results would not be possible. Data emerging from the analysis of the identified variables have therefore been aggregated and described in terms of the three identified elements of the research question (population, intervention and outcomes/study design). In addition, to obtain insights from the data, the first author also read in depth the selected articles, and performed a thematic analysis of the main emerging topics [43]. Such topics were either used to integrate and clarify the meaning of variables included in the tables, or to explore further dimensions of the interventions.

### Search results

**Figure 1:**
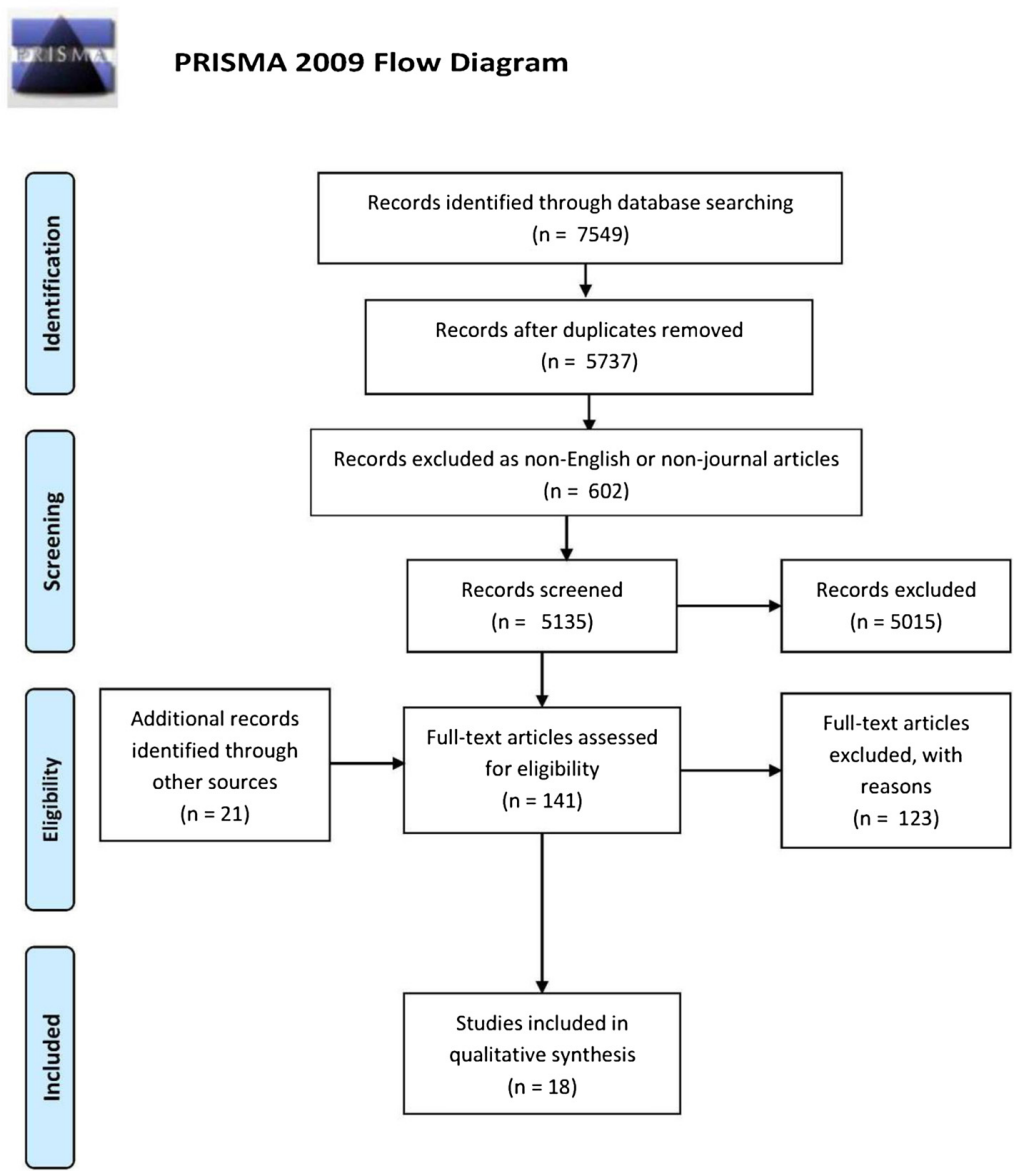
PRISMA 2009 flow diagram representing the selection process.

The PRISMA 2009 flow diagram reported in Figure 1[40] depicts the process of selection and identification of articles.

The searches in the four databases yielded 7549 references, included in a database using the reference management software Endnote X2 by Thomson Reuters. Thereafter, automatic deduplication of results was performed, followed by a manual check of all remaining references. We identified 1812 items as duplicates, while the remaining 5737 articles represented the initial dataset on which the selection was performed. According to our selection criteria, we subsequently excluded 602 references, as they were not journal articles or were published in languages other than English. We therefore conducted the screening on 5135 references. In this phase, the first author screened the title of each reference to verify if it matched the inclusion criteria. Cases of uncertainty were resolved by reading the abstract, if available. The first author resolved doubts and interpretive difficulties by discussing specific cases and criteria with all other authors.

To validate the process, two other authors (AB and EG), blinded and following the same procedures, screened 515 randomly selected references (10% of the dataset). Hence, 20% of the dataset was screened by at least two researchers: the results showed a uniform application of the screening methods. At the end of the screening process, 5015 articles were excluded, according to exclusion and inclusion criteria. Consequently, we thoroughly assessed for eligibility 120 articles, using the abstract and, in case of uncertainty, the full text. For each review identified during the eligibility assessment process, all relevant references were checked and added to the list of articles to be checked for eligibility, if not previously identified by the search strategy. Furthermore, we assessed for eligibility all citations from the included articles that had not been included in the search results. This step allowed for further inclusion of 21 articles. In total, 141 full text articles were assessed for eligibility. Eighteen matched the inclusion and exclusion criteria and were included in the review [29,44-60]. However, in some cases, more than one article reported the results of the same study, referred to different stages of the study. An intervention named "KIDS – Kids identifying and defeating stroke" was reported in three articles [29,52,56]. A stroke prevention and awareness intervention for the Hispanic community in the Chicago area was reported in two articles [48,49]. "Hip-hop stroke", which was the topic of two articles [58,59], described two entirely distinct phases of the intervention, targeted at different segments of the community and administered by different actors. Hence, they were considered as two different interventions. The total number of interventions found in the literature amounts to 15 studies, reported in 18 articles. Search and selection results also confirmed that no review was available on the topic: consequently, we classified all included articles as primary studies.

## Results

Results of the analysis are described in three synoptic tables, organized according to the P.I.C.O.S. framework, often used in systematic reviews of medical literature [40,61,62]. The acronym outlines the five main dimensions of a research question for a systematic review – population, intervention, comparison, outcome and study design. Here, we did not consider comparison, since a number of interventions did not use specific comparators: accordingly, the three tables contain a synoptic description of items referring to population, intervention and outcomes. In the tables, studies are identified by the name of the first author followed by the publication year. The complete reference is available in the final reference list. Two rows contain more than one study, since the results of more than one article were aggregated when they reported on the same intervention.

### Specific ethnic minorities, age of subjects and population sizes

Table 2 displays the main characteristics of the populations targeted by the interventions. Since all interventions took place in the US, African Americans were the most represented ethnic group, targeted by 80% of interventions, followed by Hispanics (or Mexican Americans), targeted by 53% of interventions. No other ethnic group was represented.

Most interventions were designed for groups, while 33% were delivered to individuals. However, such a distinction is sometimes difficult to establish, since in some interventions designed for groups individuals received considerable attention (e.g. by screening, individual counselling after the session, etc.) [e.g. [49,51]. No intervention targeted communities as a whole.

As for the specific population within the target ethnic groups, only 20% of interventions were targeted at either stroke or TIA patients or patients at risk for stroke. Another 20% of the interventions were delivered to the general population of a specific geographical area or community, while 60% were targeted at specific subgroups, such as church members, women, students and parents. Most interventions were targeted at adults (over 18 years of age), with only a few exceptions [50,56,58]. Further, population size proved difficult to calculate. To obtain a rough estimate, we classified the intervention as small if it involved less than 100 participants, medium if 101–500 participants were involved, and large in cases of more than 500 participants. According to this criterion, seven interventions were medium, three small and five large. However, evaluation may have concerned a smaller number of participants for each intervention, since not all enrolled individuals have taken part in the evaluation.

### Outcomes and study designs

Table 3 describes the outcomes and study design of included studies.

All interventions aimed at increasing knowledge and behavioural intention, while no intervention was specifically designed to target and measure real behavioural change directed to call EMS when witnessing a stroke.

Sixty-seven per cent of studies had an experimental design and provided some form of evaluation, while 33% of studies did not provide any evaluation [46,50,53,57,60]. However, only a minority of studies (27%) employed a randomized controlled trial (RCT) design [29,45,47], all the others being case studies. For the evaluation, eight studies used pre- and post-intervention tests [29,45,49,51,54,55,58,59], and two were only preliminary reports, lacking evaluation data [44,47].

Of the eight studies providing evaluation results, six were case studies [49,51,54,55,58,59] and two RCTs [29,45]. Both case studies and RCTs referred to very different populations, interventions and outcomes. Due to this heterogeneity (as evidenced by the synoptic tables), no quantitative synthesis of results was possible. Two studies reported that the intervention was not effective [51,55]. The six studies reporting that the intervention was effective [45,49,53,56,58,59] raise some methodological concerns. Morgenstern et al. [56] found the intervention effective only for a subgroup of the target population (school children), while insufficient data were available to establish the effectiveness of the intervention for the other subgroup (parents). Additionally, the pre- and post-intervention test was not validated. In two cases, reported in three articles [45,48,49], the difference between pre- and post-intervention test results was minimal, although statistically significant. Finally, Duraski’s study [49] had no control group. The lack of a control group also characterizes the other three studies claiming effectiveness for the described interventions [54,58,59]. Evidence of effectiveness exists in all six studies for specific outcomes in specific populations (e.g. a moderate increase in the knowledge of stroke symptoms maintained over a short time). However, no generalizable evidence of effectiveness exists for health promotion interventions aimed at improving knowledge of stroke symptoms and related actions in BME communities. All included studies used only quantitative methodology to evaluate the effectiveness of interventions, thus excluding both qualitative and mixed methods designs.

### Interventions: country, focus and delivery techniques

Tables 4 and 5 summarize the most important dimensions of the health promotion interventions described in the selected articles.

Firstly, all interventions were carried out in the United States. Consequently, all data concern a specific context, and no data on other relevant areas such as Europe, the Far East or Australia is available. All interventions included at least a component relating to awareness of stroke symptoms and related actions. Forty percent were mostly focussed on awareness, 33% had a shared focus between awareness and prevention or risk factors, while 27% were focussed on prevention or risk factors, with only few activities on awareness of symptoms. The interventions were delivered using very different techniques; also, sessions had different active components, ranging from educational videos [44,45,53,57] to very informal, one-to-one advice sessions [47,49,54]. Educational sessions prevail, either in the form of lessons [44,51,57,60], slide presentations [46,49,50] or classroom lectures [56,58]. Often, such sessions were interactive, allowing for the exchange of questions and answers between health promoters and the audience, and occasional role playing [44,49,50,54,58]. Five interventions also included distribution of informative materials about stroke, and in some interventions these materials were meant to be shared with families and friends [46,53,54,56,59]. For further details, see Table 3, column "intervention type".

### Duration of interventions, settings and actors involved

Interventions administered in a single session of information and advice were generally brief, lasting between 30 minutes and two hours. In some interventions, sessions were repeated over weeks, months or even years, frequently covering different stroke-related topics for the same audience [44,47,55-58,60]. Interventions were delivered in a range of different settings. Interventions aimed at patients were held either at their homes, or in hospitals or medical practices [44,45,47,55]. Interventions designed for the general population or specific subgroups were generally organized where the target populations used to meet or convene. Hence, churches were used for church members [46,51,53,57,60], schools for students [56,58], community centres and organizations [46,49], hospitals and medical practices [53] and one intervention was held in abeauty salon [54].

Actors designing and delivering the interventions varied widely. Forty-seven percent of interventions were delivered by multiprofessional groups, while 53% were delivered by a single profession. Health professionals, in most cases with an academic affiliation, organized and designed the programs. Interventions were delivered by nurses, research nurses and nursing students [44,49-51,55,57,60], trained health champions or ambassadors (students, church members, beauticians) [46,47,53,54,59], health educators and other community health workers [44,47,58], physicians [56,57].

### Theories underpinning interventions and techniques for cultural adaptation

We explored the extent to which the interventions were theory-based, and whether they provided clear definitions of cultural adaptation. Fifty-three percent of studies [45,49-51,53,54,58,60] did not mention the utilization of any theory to design and evaluate the intervention. As for the remaining studies it is difficult to define them as theory-based, since theories were only briefly mentioned, and no clear link with the factual content of the health promotion program was established. Four studies [44,46,56,58] mentioned social cognitive theory [63], three [44,47,55] motivational interviewing [64], three more [46,55,57] stages of change [65], two [47,58] theory of reasoned action see also [66,67], one [47] theory of planned behaviour [68]. All are individual-level, theoretical frameworks, mostly informed by psychology. Further, in most cases theories were used as a reference for stroke risk and prevention, and only in three instances [44,56,59] for the component of the intervention addressing stroke symptoms and related actions.

As for cultural adaptation of interventions, as many as 33% of the studies did not mention any specific strategy; two further studies [46,47] simply referred generically to cultural tailoring or cultural sensitivity of the interventions. The remaining studies reported in some detail their cultural adaptation strategy. These included different tactics and practical actions. Four programs [44,49,50,56] adopted of languages spoken by ethnic minorities for materials and events (especially for Hispanic communities), or used a language corresponding to the level of health literacy of community members. Three interventions [44,49,56] incorporated the point of view and specific issues of communities through focus groups or committees of community actors or workers in planning and implementation, early feedback, or community involvement. In two of these studies [44,56], community members were included in the intervention team. A related strategy was to include community-based role models as actors, artists or testimonials, and to use visuals integrating community places or surroundings [44,45,56,58,59]. Moreover, two studies [44,49] reported addressing community-specific barriers to behaviour change, and specific health beliefs and risk factors. Some consideration was also given to the social structure of communities, with related roles and cultural characteristics. "Familism", family structures and intergenerational contact were taken into account [56] and dance and hip-hop were used to convey the health promotion message [58,59].

None of these studies referred to general frameworks or models of cultural adaptation, theories of ethnicity, and the like, the approach being mostly practical. Consequently, no common definition of cultural competence or adaptation emerged from the included studies. Moreover, no study included a specific justification of the reason why some specific cultural traits had been selected as typical of that particular community.

### Barriers and facilitators for the success of interventions

No study aimed at systematically identifying barriers and facilitators for the success of the interventions, hence comments on such a topic were occasional. Only the importance of funding and continuity of the program over time was identified as a facilitator in 27% of the studies [46,51,57,60]. Other facilitators included the involvement of gatekeepers of the venues where the interventions took place [51,54,60], providing transportation [44,47,48] and financial incentives or gifts [29] and using reminders to increase participation [52]. Moreover, using small groups in interventions [51], combining a "captive audience" and a trusted educator [54] and giving participants individual attention [51] seemed to facilitate participation in some of the interventions. Conversely, barriers included time demands on gatekeepers, health professionals and coordinators [57] and the young age of some prospective participants, not perceiving stroke prevention as a priority [29].

### Community involvement: strategies and problems

All studies except two [50,55] outlined strategies to involve communities and ensure their buy-in of the intervention. Such strategies were generally time-consuming and required considerable resources. Examples included the use of committees of advocates, gatekeepers and community members, to obtain advice on community involvement, program content and delivery techniques and channels and on the final evaluation. Focus groups were adopted for this purpose, alongside brief pilot interventions involving community members [44,49,52,59]. Particular care was taken in identifying community gatekeepers to help or take the lead in organizing sessions [48,51,54,57,60], while some interventions explicitly adopted a train-the trainer approach [46,47,53,54,57,60]. In this case, health professionals and campaigners provided stroke education to specific members of the community, such as school children, pastors, beauticians, who, in turn, played a relevant part in educating parents, customers or church attendants. In the case of a particularly sizeable and structured program, the whole organization of sessions in a community site (association, church, etc.) was devolved to previously instructed local leaders [57]. Other involvement strategies included fostering flexibility and community creativity [52,57], using community-based health professionals [29,57], and adopting multi-channel involvement strategies (word of mouth, gatekeepers, web site, advertising, community association meetings) [29,57].

No intervention was aimed at specific social networks within communities. When sizeable groups were targeted, the main objective was still to increase the knowledge of individuals within the groups, without consideration for community or social network dynamics. Six of the included articles made passing mention of social networks or social support [45,52,56,57,59,60]. Some studies mentioned the importance of using schoolchildren as trainers for their parents, regarding stroke knowledge, and underlined the related difficulties [56,59]. Others generically mentioned the importance of improving stroke knowledge of relatives and other members of the support networks for stroke patients [45,52]. The most complex intervention stressed both the importance and the difficulty of coordinating networks of different organizations involved [57]. In the same study social ties and networks were used to recruit participants for the intervention, and to plan, test and implement it. Collaboration between networked actors with different roles was highlighted as important for the success of a further intervention [60]. However, none of the interventions put in place a systematic strategy for tapping social networks and social support resources.

### Educational strategies adopted in the interventions

Ten studies emphasized the value of active, interactive and experiential education strategies, but only some of these provided details of the educational methods. Small groups [29,57], role-plays and enactment of scenarios by participants [29,44,59], encouraging flexible discussion of stroke awareness [29,44,56-58], enabling self-efficacy [29,52,56] and interactive multimedia resources [29,52,56,58] were the most frequently cited methods. Exercises and assignments were used to promote involvement of participants in producing health promotion materials and spreading the message to their families [29,52,56,57,59]. Arts and music were also employed to involve community members in the educational activities, together with promoting the creativity of participants [29,52,56,58,59]. In long interventions [29,52,56-60], different methods were linked to each other and produced positive feedback. For example, in one circumstance, students interviewed their parents on stroke awareness and then produced information materials accordingly [29,56].

## Discussion

Community-based health promotion (CBHP) interventions are usually considered complex and difficult to plan, perform, and evaluate. This is due to the high number of variables involved, including complex behavioural factors, the influence of culture and norms on health behaviours and the simultaneous presence of several health promotion interventions [69,70].

This difficulty is clearly revealed by the studies included in our review. Firstly, even in a relatively homogeneous context of community-based health promotion interventions aimed at African Americans or Hispanics living in the US, planning and evaluation methods differed significantly. There is growing recognition that the design and evaluation of health promotion interventions need to be informed by theory [71-73]. However, theory-based health promotion interventions were in the minority in the sample used for our review. Consequently, clearly specifying the theoretical foundations of the design and evaluation methods of an intervention may considerably improve its implementation.

Moreover, theories used in the included studies focussed on the individual level, while six studies have recently showed that community- and network-level theories may prove effective in designing and evaluating community-based health promotion interventions [74-79]. Hence, using community- or network-oriented theories may help health promoters to systematically take into account dimensions of an intervention that could have a significant impact on its effectiveness.

Furthermore, no evidence was available for European countries, where minorities show cultural and social characteristics directly influencing health-related attitudes differing from US minorities. For example, Scheppers et al. [80] show that ethnic minorities experience a number of barriers to accessing health services, frequently linked with particular cultural, religious or social practices. Such practices are different between ethnic minorities: for example, UK Pakistanis and US African-Americans are likely to have very different health beliefs and ill health-attribution. Scheppers et al. also maintain [80], that the organization of health services in different countries plays an important contextual role in shaping health-related attitudes of ethnic minorities. Also, studies concerning the definition of cultural competence often emphasize the importance of specific, contextual aspects, rather than abstract definitions of ethnicity, in order to successfully conduct health promotion and health care initiatives [38,81]. Therefore, specific key factors (perception of health and illness, language, available community resources, specific barriers and facilitators) may greatly differ between different ethnic minorities living in different countries. For all these reasons, it seems difficult to generalize any results from the included studies to the UK or European situation. It would, however, be important to note that further research related to stroke awareness improvement is aimed at addressing non-US based ethnic minorities.

Although in most studies cultural adaptation was considered as important, cultural adaptation was almost always linked to practical or pragmatic strategies. Consequently, no intervention took into account the recent contributions that tried to define cultural appropriateness of interventions and the main principles for conducting research on ethnicity and health [37-39]. Consequently, using theory and data from this specific literature may help in designing interventions that are more congruent with specific characteristics of ethnic minorities.

Furthermore, the evidence of effectiveness emerging from the included studies is inconclusive. Fifty-three percent of the studies provided evaluation results, and only 25% of these included a control group. As a result, current available evidence of the effectiveness of such interventions seems inconclusive. In addition, no qualitative study satisfied our inclusion criteria. While acknowledging the importance of quantitative evidence, it seems that integrating a qualitative approach would be appropriate for complex health promotion interventions. In fact, such interventions involve different variables such as ethnicity, knowledge and behaviour change, and - most importantly – their success seems to heavily depend on complex contextual factors. Qualitative research is often advocated as an appropriate method in evaluating health promotion interventions [82,83] especially because it can provide an holistic perspective [84,85]. Qualitative research may therefore explore this under-researched topic and identify dimensions influencing the effectiveness of stroke awareness interventions for ethnic minorities.

### Strengths and limitations

To the best of our knowledge, this was the first systematic review to date on health promotion interventions for stroke awareness in ethnic minorities. The results outline the main characteristics of stroke awareness interventions for ethnic minorities in the US, alongside strength and limitations of both the interventions and the evaluation procedures. Review findings may therefore provide a useful starting point for academics and practitioners wishing to further analyse or plan similar health promotion initiatives in other parts of the world.

Limitations include the fact that searches were restricted to peer-reviewed journal literature written in the English language. Although we searched the most relevant databases, broadening the search to supplementary sources and including more languages may increase the number of retrieved studies. The same results might be obtained by the inclusion of conference proceedings, dissertations, books and book chapters and grey literature results.

### Implications for further research

Since cultural adaptation seems important in this context, there is a critical need for studies on health promotion interventions for stroke awareness in ethnic minorities other than African Americans and Hispanics.

Furthermore, such interventions operate in very complex scenarios, and several variables may have an impact on their effectiveness. Qualitative or mixed-methods study designs may help to understand contextual factors influencing community-based health promotion, including barriers and facilitators, experiential education strategies and methods for involving communities.

Finally, network- and community-level health promotion theories may contribute useful insights both in designing and evaluating health promotion interventions on stroke awareness for ethnic minorities.

## Conclusions

In this review we set out to find evidence about the effectiveness of interventions to increase stroke awareness in ethnic minorities. Our results show that this is a particularly understudied area, and that all included studies refer to the US.

Evidence of effectiveness from 11 case reports and four RCTs focussed on short- to medium-term knowledge improvement for individuals seems particularly weak and inconclusive. Therefore, we suggest that further research is conducted on different countries and ethnic minorities.

## Endnote

^a^The acronym of the campaign stands for Face, Arms, Speech, Time to call EMS (the first three being distinctive stroke symptoms).

## Competing interests

The authors declare that they have no competing interests.

## Authors’ contributions

All authors contributed equally in designing the study and critically discussing the results. PG carried out the bibliographic searches, selected the studies, analysed the data, contributed to the discussion and drafted the manuscript. EG and TR contributed to the study design, selection of studies and interpreting the results. AB contributed to design the search strategies, the selection process and the interpretation of results. EG, TR and AB critically revised the manuscript. All authors read and approved the final manuscript.

## Pre-publication history

The pre-publication history for this paper can be accessed here:

http://www.biomedcentral.com/1471-2458/14/409/prepub
